# Elevation of Tear MMP-9 Concentration as a Biomarker of Inflammation in Ocular Pathology by Antibody Microarray Immunodetection Assays

**DOI:** 10.3390/ijms23105639

**Published:** 2022-05-18

**Authors:** Miguel de la Fuente, Iñaki Rodríguez-Agirretxe, Elena Vecino, Egoitz Astigarraga, Arantxa Acera, Gabriel Barreda-Gómez

**Affiliations:** 1 Department of Research and Development, IMG Pharma Biotech S.L., 48160 Derio, Spain; miguel@imgpharma.com (M.d.l.F.); egoitz.astigarraga@imgpharma.com (E.A.); 2 Experimental Ophthalmo-Biology Group (GOBE), Department of Cell Biology and Histology, University of the Basque Country UPV/EHU, 48940 Leioa, Spain; elena.vecino@ehu.es; 3 Department of Ophthalmology, Donostia University Hospital, 20014 San Sebastian, Spain; ira@icqo.org; 4 Begiker-Ophthalmology Research Group, BioCruces Health Research Institute, 48903 Barakaldo, Spain; 5 IKERBASQUE, Basque Foundation for Science, 48009 Bilbao, Spain

**Keywords:** tear MMP-9, enzyme biomarker, diagnosis, monitoring, antibody microarray, ocular inflammation, glaucoma, point of care, in vitro diagnostics

## Abstract

Matrix metalloproteinases are a family of enzymes fundamental in inflammatory processes. Between them, MMP-9 is up-regulated during inflammation; thus, its quantification in non-invasive fluids is a promising approach for inflammation identification. To this goal, a biomarker quantification test was developed for ocular inflammation detection using anti-MMP-9 antibody microarrays (AbMAs). After validation with eight healthy control tear samples characterized by ELISA, 20 samples were tested from individuals diagnosed with ocular inflammation due to: cataracts, glaucoma, meibomian gland dysfunction, allergy, or dry eye. Concentration values of tear MMP-9 were obtained for each sample, and 12 patients surpassed the pathological threshold (30 ng/mL). A significant elevation of MMP-9 concentration in the tears of glaucoma patients compared with healthy controls was observed. In order to evaluate the diagnostic ability, an ROC curve analysis was performed using our data, determining the optimal threshold for the test at 33.6 ng/mL of tear MMP-9. In addition, a confusion matrix was applied, estimating sensitivity at 60%, specificity at 88%, and accuracy at 68%. In conclusion, we demonstrated that the AbMAs system allows the quantification of MMP-9 in pathologies that involve inflammation of the ocular surface.

## 1. Introduction

Biomarkers can be defined as biological analytes by which a particular pathological or physiological process can be identified or characterized [1]. They allow a more precise diagnosis and the monitoring of pathologies and conditions. A biomarker can determine if the patient has a particular medical state, the different subtypes of the pathology if applicable, and the best treatment indicated, improving the monitoring of the therapy response, the diagnosis, and the progression [2].

Among all types of biomarkers, enzymes are gaining importance in many pathologies [3,4]. Enzymes are chemical catalysts that help organisms conduct essential biochemical reactions. Deficiency, malfunction, reduced/increased activity, or overexpression of enzymes and their inhibitors can cause a variety of clinical conditions [5]. Consequently, the study of enzymes and their inhibitors is cardinal for understanding disease pathophysiology and developing not only therapeutic options but also diagnostic and monitoring strategies, as enzymes are powerful markers of disease [5,6]. In this regard, detection and quantification of enzymes in biological fluids is an interesting field of research, as it can lead to improvements in pathology prognosis and patient life.

One of the main processes in which enzymes participate is inflammation, a fundamental mechanism for maintenance of body homeostasis versus infections and injuries. Novel published research has established a relationship between systemic inflammation and several highly prevalent pathologies, such as cancer [7] and neurodegenerative [8], autoimmune [9], cardiovascular [10], and metabolic diseases [11]. In addition, many ocular pathologies such as Sjogren’s syndrome [12], or keratoconjunctivitis sicca, commonly named as dry eye (DE) [13], have also been correlated with inflammation. Furthermore, antimicrobial preservative compounds such as quaternary ammonium benzalkonium chloride (BAK), used in antiglaucoma eye drop treatments, have been associated with chronic ocular inflammation [14,15]. Many clinical symptoms of chronic ocular inflammation have been reported in patients under long-term antiglaucoma treatment [16]. It has been determined that BAK acts at different levels of the cell machinery, interacting with cell membranes and receptors. It affects conjunctival epithelial cells and provokes ocular inflammation signs and symptoms such as loss of goblet cells, conjunctival squamous metaplasia and apoptosis, disruption of the corneal epithelium barrier, and damage to deeper ocular tissues [16]. These toxic effects trigger inflammation pathways that precipitate the overexpression of certain enzymes. Taking this into account, enzymes can be used as biomarkers, either for diagnosis or for monitoring the response to a treatment and evaluating the adverse and toxic effects of the therapy.

Matrix metalloproteinases (MMPs) are a family of enzymes that play important roles in inflammatory processes [17,18]. MMP-9, also called gelatinase B, is a zinc and calcium ion-dependent enzyme that is involved in tissue remodeling by degrading types IV and V collagen of the extracellular matrix (ECM) in physiological processes such as wound healing and bone growth [19,20]. This enzyme plays an important role and is upregulated in inflammatory pathologies, arthritis, cardiovascular and pulmonary diseases, as well as in cancer [18]. MMP-9, along with other MMPs, is upregulated during inflammation in different tissues and fluids such as serum, saliva, synovial liquid, or tear, becoming an interesting enzyme biomarker. Thus, detection and quantification of MMP-9 in non-invasive fluids is a promising approach for inflammation prevention, diagnosis, and disease or treatment monitoring. Concretely, MMP-9 has been also extensively studied as a biomarker of inflammation in tear samples [21,22,23,24,25]; this biomarker is highly overexpressed in different diseases associated with ocular inflammation and in ocular surface pathologies [22,26,27]. In the corneal epithelium, both TGF-β and IL-1 cytokines, key players in the regulation of inflammatory processes, stimulate MMP-9 overexpression [28].

Currently, the diagnosis of the main ocular surface inflammation pathologies is mostly subjective and is based on the knowledge of the ophthalmologist and the signs and symptoms of the patients [29,30]. However, the discovery and use of biomarkers, such as MMP-9, have opened new lines of research aiming to develop new diagnosis tools [31]. These biomarkers are extremely useful in the clinic because they reflect the pathological state of the patient, as well as the evolution of the disease in molecular terms; thus, they can be used, not only for diagnosis but also for evaluating the prognosis and for monitoring the progression of the pathology and the response to treatments. Various studies have validated tear MMP-9 as one of the main biomarkers for ocular inflammation diseases [23]. Different commercial diagnosis point of care (PoC) and in vitro diagnostics (IVDs) tests have been developed for evaluation of tear MMP-9, such as InflammaDry [24,27]; however, these types of tests have the main drawback of giving only a positive/negative result, not allowing the quantification of the biomarker, nor a precise evaluation of the pathological status of the patient, nor the monitoring of the disease, due to their variability [32]. Additionally, this hampers the correlation between symptoms and biomarker concentration, precluding the stratified diagnosis of patients.

Alternatively, microarray technology can be applied as a platform for biomarker-based diagnostics or monitoring. Cell membranes, whole cells, antibodies, enzymes, nucleic acids, and other proteins can be immobilized on diverse surfaces using microarray technology without losing their functional structure. As a result, they are used in immunochemistry, autoradiography, radioligand and binding investigations, mitochondrial toxicity assays, as well as other techniques such as colorimetry and mass spectrometry [33,34,35,36,37]. Microarrays allow the reduction of the number of samples, medications, chemicals, and residues. Among them, antibody microarrays (AbMAs) are used similarly as a miniaturized enzyme-linked immunosorbent assay (ELISA) for the detection of analytes. However, AbMAs show higher sensitivity for the identification of biomarkers than traditional ELISA, demonstrating their improvements in clinical situations when taking also into account the previously mentioned advantages [25]. Currently, AbMAs are widely used for disease diagnosis in diverse pathologies such as cancer [38], or ocular conditions [39], among others [40,41].

**Figure 1:**
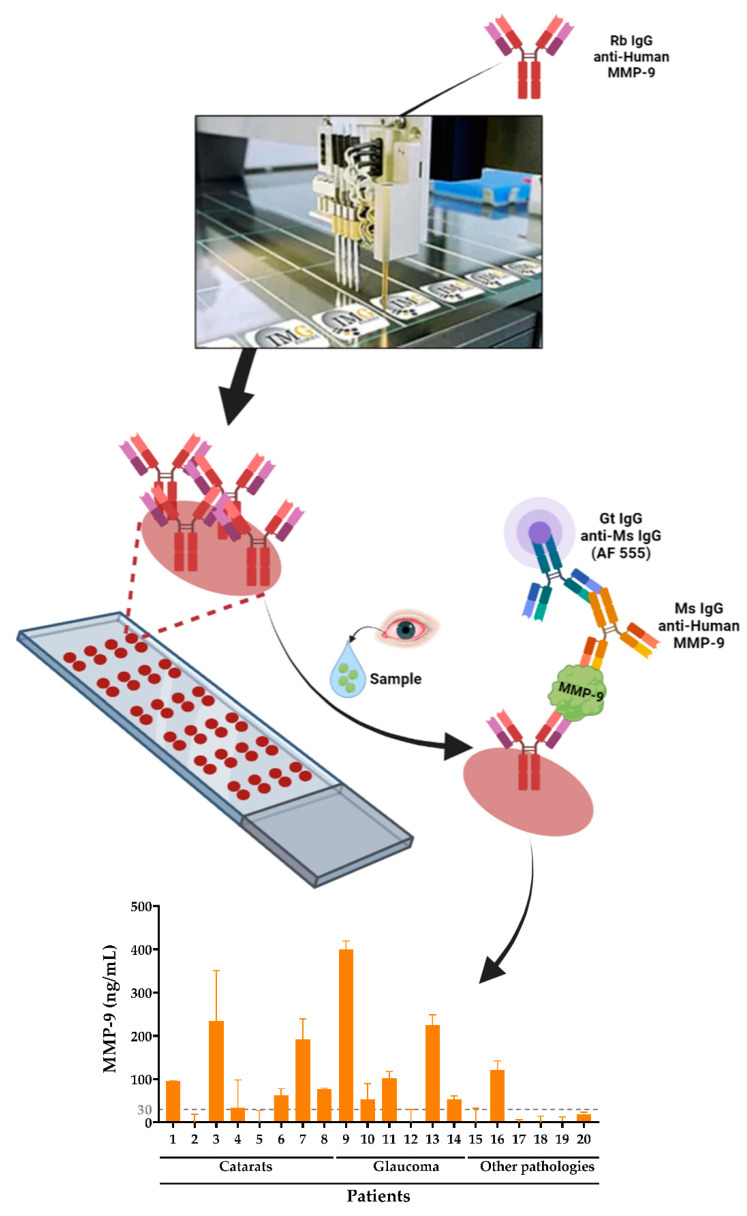
Detection and quantification of MMP-9 enzyme inflammation biomarker in human tear samples using AbMAs. First, the selected antibodies were immobilized onto glass slides that were incubated with the sample. Then, MMP-9 was captured by the mentioned antibody and detected with a labeled antibody cocktail. Finally, the intensity of the signal was quantified and the data acquired, allowing the analysis of the MMP-9 biomarker in the samples.

Hence, the aim of this work was to develop an AbMA test for ocular inflammation detection by quantifying tear MMP-9 biomarker (Figure 1). For this purpose, antibodies against human MMP-9 were immobilized over glass slides where the sample was incubated and the biomarker was captured. Then, the biomarker was detected using a labeled antibody cocktail that produced a fluorescent intensity signal directly proportional to the concentration of MMP-9 in the sample. For the validation of the test, eight non-pathological tear samples were used. Enzyme MMP-9 biomarker concentration was confirmed using conventional ELISA as the gold standard to characterize the samples and assess the reliability of the test. Subsequently, tear samples from 20 individuals clinically diagnosed with ocular inflammation were assayed. Using a calibration line, protein biomarker presence was quantified in each of the samples employing AbMAs, early validating the developed technique as an MMP-9 inflammation-related detection tool.

## 2. Results

### 2.1. Subjects

A cohort of samples from both healthy controls and patients suffering ocular inflammation was used to validate customized AbMAs as an MMP-9 quantification assay for ocular inflammation evaluation in human tear fluid.

Firstly, tear samples were obtained from volunteers as detailed in the Materials and Methods Section. Tear samples were divided into two groups: healthy controls, named as HC, and patients, named as P. The second one was composed of individuals suffering ocular inflammation due to different pathological conditions such as cataracts, glaucoma, meibomian gland dysfunction (MGD), allergy, or DE. Patients were evaluated using a Schirmer’s test; normal values were considered ≥10 mm wetting of the paper after 5 min, whereas tear deficiency values were ≤5 mm. All patients, except from P 9, P 11, and P 18, presented tear deficiency. Glaucoma patients were all under prostaglandin eye drop treatment; these drugs were preserved with BAK. P 9 was under two different BAK-preserved prostaglandin analogue treatments. No Schirmer’s test was performed for healthy volunteers to avoid the intervention since they did not present clinical conditions. In addition, the gender and age of the patients were detailed (Table 1).

### 2.2. Antibody Microarray Validation

**Figure 2:**
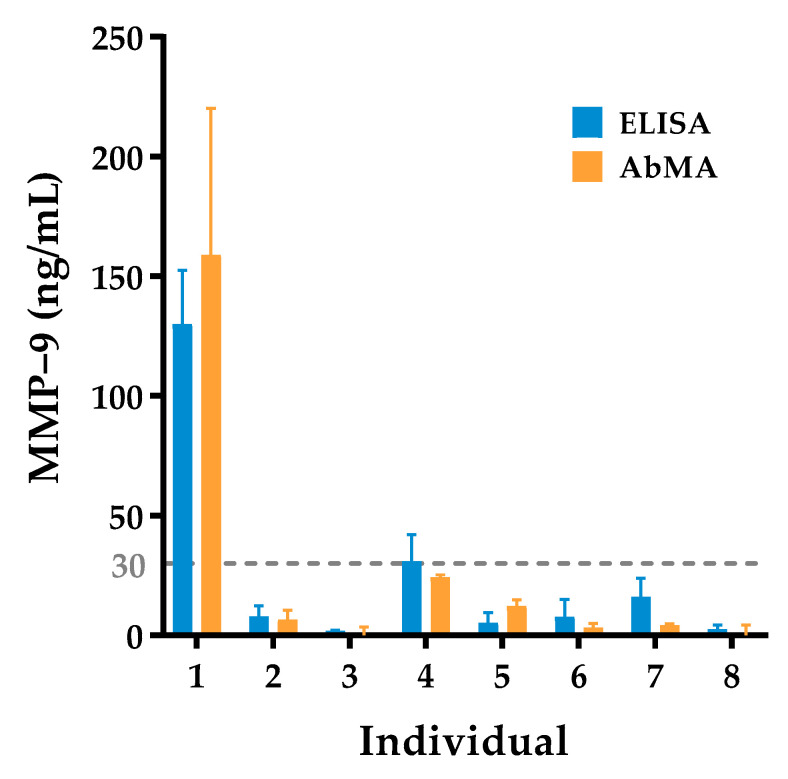
Concentration of MMP-9 in the collection of tear samples from healthy controls, without any ocular disorder diagnosed, using ELISA (blue) as the gold standard technique and AbMAs (orange). MMP-9 concentration is represented as ng/mL for each individual. A gray line is plotted at 30 ng/mL of MMP-9 enzyme in tear, the threshold value at which higher concentrations are considered a sign of ocular inflammation.

The eight samples from the healthy volunteers were characterized using an anti-human MMP-9 ELISA kit. In order to assess the reliability of this technique, in contrast with the gold standard, the obtained values were compared with the quantification of tear MMP-9 using the developed AbMAs. When comparing the concentration of MMP-9 obtained with each technique, similar results were obtained (Figure 2). Additionally, a simple bivariate correlation was calculated, setting the significance at α = 0.05 using a two-tailed test. A Pearson correlation coefficient (r) of 0.9918 was obtained with a significance of (****), *p*-value < 0.0001.

### 2.3. Analysis of Pathological Samples

**Figure 3:**
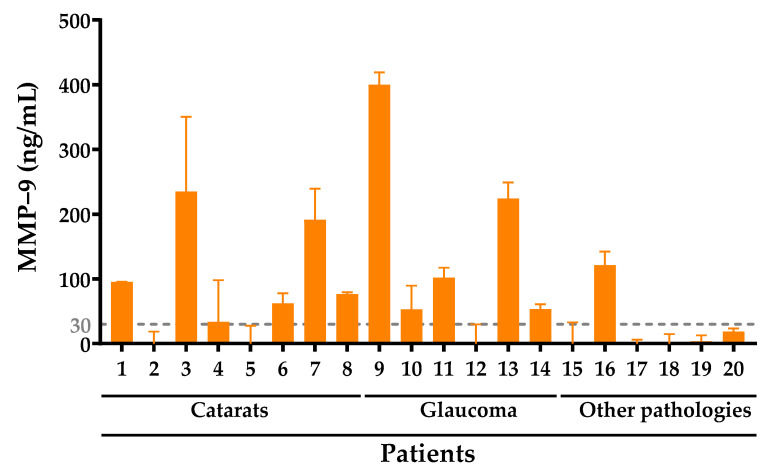
ng/mL of MMP-9 in ocular inflammation patient tear samples quantified using AbMA. A gray line is plotted at 30 ng/mL of MMP-9 enzyme in tear, the threshold value at which higher concentrations are considered a sign of ocular inflammation.

Enzyme biomarker MMP-9 was also evaluated in the 20 patient samples using the AbMA developed technology. Concentration values of MMP-9 were obtained for each tear sample. P 1, P 3, P 4, P 6, P 7, P 8, P 9, P 10, P 11, P 13, P 14, and P 16 samples surpassed the pathological threshold established at 30 ng/mL of MMP-9 in the fluid. These 12 samples represent 60% of the tear collection from patients diagnosed with an ocular pathology used in this study (Figure 3).

**Figure 4:**
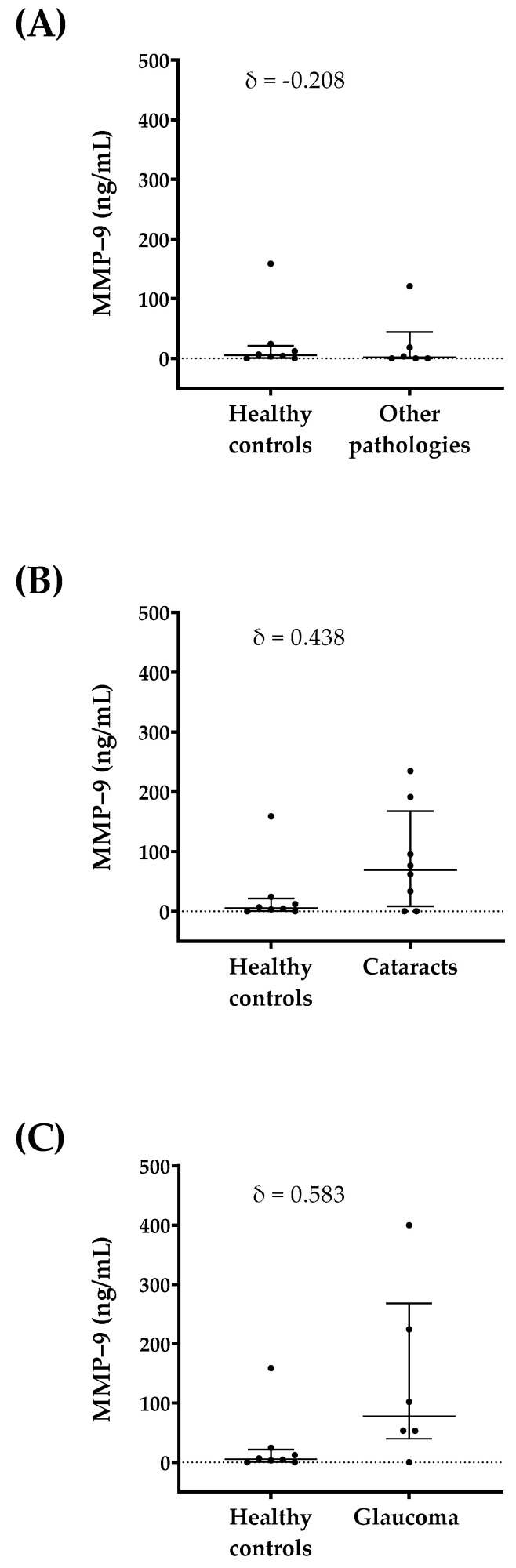
Tear MMP-9 concentration differences in the groups of patients suffering ocular inflammation versus the group of healthy controls. Cliff’s delta values are displayed for each comparison. (**A**) Differences between healthy controls and MGD, DE, and allergy patients (other pathologies group). (**B**) Differences between healthy controls and cataracts patients. (**C**) Differences between healthy controls and glaucoma patients.

In addition, the MMP-9 concentration in each tear sample was compared between healthy and pathological subgroups (Figure 4). The normality of the samples was evaluated using a Shapiro–Wilk test; setting the significance at α = 0.05 using a two-tailed test, the groups did not follow a Gaussian distribution. The patient group was divided into a glaucoma group, a cataract group, and the other pathologies group, englobing MGD, allergy, as well as DE. Cliff’s delta values were calculated for quantifying the amount of difference between control and pathological groups. The Cliff’s delta value when comparing the healthy group and the other pathologies group was δ = −0.208; for cataracts patients versus healthy individuals, it was δ = 0.438; and for glaucoma patients versus healthy individuals, it was δ = 0.583.

No differences were observed when comparing tear MMP-9 concentrations between age, gender, and Schirmer’s test results (data not shown).

**Figure 5:**
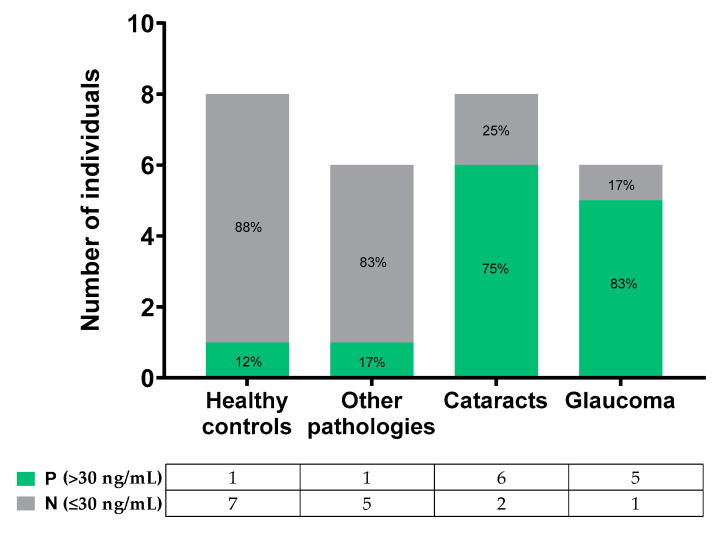
Number of individuals or patients over and below the pathological threshold in the different groups.

When analyzing the inflammation biomarker in the samples based on the established pathological threshold (30 ng/mL), differences were observed between the groups (Figure 5). Both healthy controls and other pathologies groups presented MMP-9 concentrations mainly below the threshold; contrarily, the cataracts and glaucoma groups presented tear MMP-9 values mostly over 30 ng/mL. In summary, 88% of the healthy controls and 83% of the other pathologies group samples were under the threshold; 75% of cataracts and 83% of glaucoma tear samples were over the threshold.

### 2.4. Evaluation of the Diagnostic Performance of the Test

**Figure 6:**
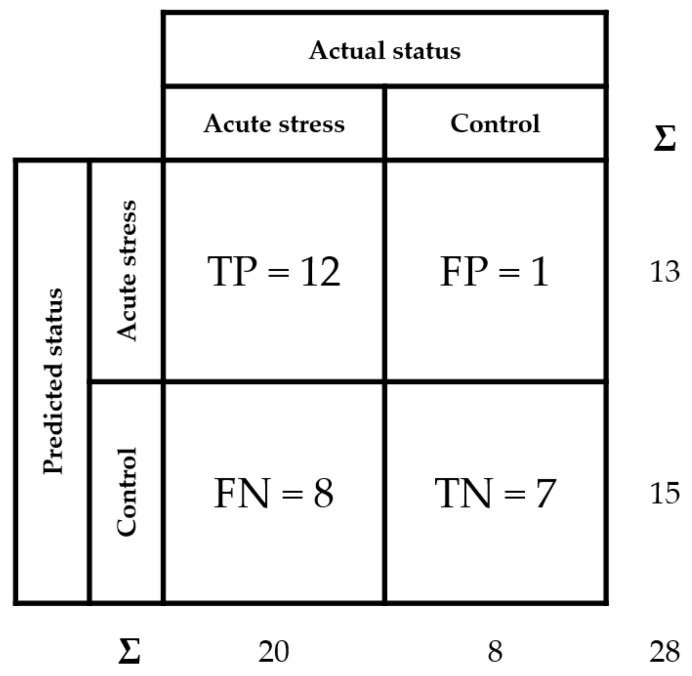
Confusion matrix of tear MMP-9 analysis over the different samples. True positive (TP), false positive (FP), false negative (FN), and true negative (TN) rates are detailed. Sensitivity is calculated as TP/(TP + FN), specificity as TN/(TN + FP), and accuracy as (TP + TN)/(TP + FP + FN + TN).

Finally, in order to evaluate the diagnostic ability of the developed AbMA test, a receiver operating characteristic (ROC) curve analysis was performed [42,43]. The optimal threshold value for the test was determined using this analysis, resulting in 33.6 ng/mL of tear MMP-9 using our data. In addition, a confusion matrix was set up (Figure 6) following the indications of the guide The Fitness for Purpose of Analytical Methods of Eurachem [44], assessing the sensitivity at 60%, the specificity at 88%, and the accuracy at 68%.

## 3. Discussion

We developed an AbMA test immobilizing an anti-MMP-9 IgG antibody and establishing a detection protocol for the quantification of MMP-9 in tears. The purpose of this test is the detection and quantification of human MMP-9 in tear samples as an instrument for ocular inflammation evaluation. The data obtained demonstrated once again that MMP-9 is a good biomarker of inflammation in various ocular pathologies [45], as well as validating microarray immunodetection technology as a diagnostic tool in the detection of MMP-9 and the monitoring of patients with inflammation-related pathologies such as glaucoma.

Our results validated the developed AbMA test for the detection and quantification of human MMP-9 in tear samples as an instrument for ocular inflammation evaluation. For this purpose, eight tear samples from healthy individuals were collected, as well as 20 tear samples from patients suffering various ocular inflammatory conditions: cataracts, glaucoma, meibomian gland dysfunction, allergy, and DE. All 28 tear samples were defined in terms of donor age, gender, condition and Schirmer’s test results. Firstly, MMP-9 concentration was determined with the currently used gold standard in this area, the ELISA technique, in order to evaluate if there is a positive correlation between this technique and the AbMA test, since the AbMA test aims to be a new method for biomarker quantification. The concentration of MMP-9 in the tear collection from healthy donors was quantified by ELISA and AbMAs according to their specific protocols. A statistically significant correlation in the obtained values was observed, with a Pearson correlation coefficient of 0.9918 and a *p*-value < 0.0001. These results are in agreement with previous studies using this technology in human tear [25], pointing out that AbMAs are a useful and reliable tool for MMP-9 quantification in human tear samples.

Once the test was validated, tear samples of 20 patients with ocular inflammation were studied. These patients were suffering from different diseases related to inflammation (cataracts, glaucoma, MGD, allergy, and DE) [46]. MMP-9 biomarker was quantified using the developed AbMAs, which resulted in 60% of the samples surpassing the pathological threshold. This was established at 30 ng/mL based on the literature [47]; higher concentrations of the enzyme biomarker in tear samples were associated with ocular surface inflammation [26]. The observed global elevation of MMP-9 in the pathological samples was reasonable due to the inflammatory characteristics of this biomarker, which increases in response to stress when cytokine or chemokine pathways are activated. When MMP-9 values were analyzed for each subject within the four study groups, the MMP-9 threshold was exceeded by only 12% and 17% of individuals in the control and other pathology groups, respectively. However, 75% and 83% of patients in the glaucoma and cataract groups, respectively, had a tear MMP-9 concentration above the pathological threshold of 30 ng/mL, denoting ocular surface inflammation. Similar results were described by Kim and coworkers when they reported that approximately 72% of glaucoma patients displayed a high concentration of tear MMP-9 (over 40 ng/mL); however, when studying a control group of 47 healthy subjects, only about 32% of them showed an increase in this biomarker [48]. Again, this validated the AbMA results in concordance with the literature [25], highlighting the importance of MMP-9 as a tear biomarker of ocular surface inflammation.

In order to assess the diagnosis capability of the test when studying each pathology, tear MMP-9 concentration was compared among groups and Cliff’s value was calculated as a useful complementary analysis for the corresponding hypothesis testing [48]. When compared with the healthy controls, the other pathologies, cataracts, and glaucoma groups obtained δ values of −0.208, 0.438, and 0.583, respectively. Values over a δ = 0.474 meant a large difference between the two groups [48]. Taking this into account, the glaucoma group was the one with the highest δ when compared with the controls, which indicated a major difference in the presence of this biomarker, preliminarily pointing out that the developed technology was able to detect ocular inflammation pathology-related states. Likewise, the cataracts group presented a δ = 0.438, meaning a medium (defined at the interval 0.330–0.474) difference between tear MMP-9 in the cataracts group and healthy controls.

Afterwards, an ROC curve analysis was performed in order to validate the reliability of the AbMA test [42,43]. The method used for determining the optimal threshold was to calculate the distance of each cut-off point to the point (0, 1) on the upper left-hand corner of the ROC space. At this point, sensitivity was 100% and 1-specificity was 0%; thus, among our data, the closest value of the ROC curve to this point was the best threshold [42,43]. We determined this value at 33.6 ng/mL of tear MMP-9, according to the pathological threshold of this biomarker presence established in the literature [26,47]. In addition, a confusion matrix was set up for the characterization of the AbMA [44]. Sensitivity, specificity, and accuracy were calculated, obtaining values of 60%, 88%, and 68%, respectively. Remarkably, high values of specificity reflected a precise ability of the AbMA test to correctly identify people without the disease, thus avoiding the administration of a treatment to a healthy person. The presented results preliminarily validated the functionality of the AbMA assay, not only in quantifying MMP-9 but also in being able to monitor the inflammatory response resulting from BAK-preserved eye drop treatments in glaucoma patients. The functionality and reliability of the developed AbMAs were demonstrated for MMP-9 quantification in tear fluid.

The possibility of objectively and quantitatively measuring enzyme biomarkers, such as MMP-9, in tear samples, will help ophthalmologists to perform more precise diagnoses, individually administer an adequate treatment, and be able to monitor the response of the patient [49]. The AbMA specific technology for biomarker detection is capable of analyzing small volumes of tear fluid [50], an important characteristic when assaying tear samples from patients with ocular inflammation, which lack tear fluid. The AbMA test needs only 2 µL of tear, diluted 1:10 in 20 µL of buffer, to measure the concentration of MMP-9 in the sample, the main advantage when compared with the alternative biomarker quantification technique, ELISA, in which the wells are usually filled with 100 µL of diluted sample. This technology, when applied to glaucoma patients, permits the monitoring of their inflammatory response to treatments, commonly related to BAK preservatives. In addition, our data provide the first quantification of tear MMP-9 in glaucoma patients, generally measured by semi-quantitative methods such as InflammaDry, which generates a positive result when tear the MMP-9 concentration is over 40 ng/mL [51,52], or by zymography, a technique that evaluates the activity of the collagenases in a sample [53]. These improvements will translate into additional benefits for patients and physicians, as well as for healthcare systems.

An explanation should be added for the special increase in MMP-9 in tear samples of glaucoma patients. This biomarker’s increase in glaucoma, together with the elevated δ value, can be associated with the pro-inflammatory adverse effects of using BAK as a preservation agent in prostaglandin eye drop treatments. Prostaglandin acts by lowering intraocular pressure (IOP), the major risk factor for glaucoma [51]. Nevertheless, even though both preservative-free eye drops and BAK-preserved prostaglandin analogues showed a decrease in IOP [54], many studies have confirmed the toxicity of using BAK preservatives, which lead to destabilization of the precorneal tear film, disrupting the mucin layer and increasing tear osmolarity, which provokes DE and ocular surface disease (OSD) progression [55,56,57,58].

BAK-preserved eye drop prostaglandin analogues trigger the expression of inflammatory cytokines [59] that provoke the augmentation of MMPs in the tear fluid [60]. We hypothesize that this response explains the augmentation of MMP-9 observed in this work. The six patients suffering glaucoma whose tear samples were assayed in the study were under BAK-preserved prostaglandin treatments and presented an elevation of the biomarker concentration. Contrarily, these differences observed between glaucoma patients and healthy individuals were not noticed when studying the other pathological groups. This hypothesis is strengthened when taking notice of P9, the glaucoma patient with the highest value of MMP-9 concentration in tear, who was under the treatment of two different BAK-preserved prostaglandin analogues. This major exposure to BAK may lead to a more considerable inflammatory response, reflected in a higher presence of biomarkers such as MMP-9. It has been considered that the cytokine inflammatory response mediated by BAK triggers an imbalance between MMP-9 and its inhibitor, TIMP-1, increasing the presence of the metalloproteinase and decreasing the TIMP-1 concentration [61]. Glaucoma development has been related to elevated concentrations of ocular MMPs [62]. In the eye, the turnover of the ECM at the trabecular meshwork is mediated by these enzymes, controlling outflow resistance and helping to maintain IOP homeostasis. An imbalance between MMPs and their inhibitors can be involved in augmentation of the IOP and trigger glaucoma [62]. This may explain long-term anti-glaucoma treatment failures and the worsening of the pathology due to elevated concentrations of MMPs.

Nowadays, glaucoma is diagnosed and monitored by recognizing morphological alterations in the optic nerve head and in the retinal nerve fiber layer caused by the loss of retinal ganglion cells. Assessment of visual function is also central to glaucoma diagnosis and pathology tracking [63]. In recent years, new ocular imaging devices, as well as structural and functional tests, have been implemented in order to improve the diagnosis and monitoring; nevertheless, these approaches are both based on evaluating the consequences of the visual damage caused by the pathology [64]. Thus, early diagnosis and monitoring tools are needed in order to avoid reaching these conditions. In this sense, the evaluation of enzyme biomarkers in non-invasive fluid samples will allow a better follow-up of the disease, which will conclude with an improvement of the prognosis [65]. Taking this into account, it is fundamental to be able to monitor the ocular inflammatory response of glaucoma patients, particularly under BAK-preserved prostaglandin treatments. Early detection of the elevation of certain biomarkers, such as MMP-9, could be the first sign of adverse toxic effects of the therapy and indicate the necessity of a change in the doses, the drug, or the treatment.

## 4. Materials and Methods

### 4.1. Tear Samples

A total of 20 patients and eight healthy control volunteers were enrolled. This research was performed by medically qualified personnel after approval by the institutional review board of the Hospital Universitario Donostia (San Sebastian, Spain) and in strict accordance with the tenets of the Declaration of Helsinki. Patients were recruited from the Hospital Universitario Donostia. Informed consent was requested of all patients after an explanation of the nature and possible consequences of the study. SARS-CoV-2 diagnostic tests were performed on each individual before sample extraction. Tear samples were collected from the inferior lateral tear meniscus, minimizing irritation to the ocular surface or lid margin. Anesthetic drops were not instilled. Tear samples were obtained by using Blaubrand microcapillary tubes from intraMark (#7087-09, Wertheim, Germany). After collection, tear samples were introduced into 0.5 mL tubes from Eppendorf (#40420050, Hamburg, Germany) and stored at −80 °C until analysis. The patients included in this study were suffering ocular inflammation due to different pathological conditions. The patients complained of eye symptoms such as foreign body sensation, epiphora, pain, or irritation. Within the patients’ groups, the individuals suffering cataracts were awaiting surgery. No clinical tests were performed on the day of tear collection, in order not to interfere with tear composition. A standard Schirmer’s test with topical anesthesia was performed by placing a sterilized strip of Schirmer-Plus Gecis (Neung sur Beuvron, France) in the lateral canthus away from the cornea and left in place for 5 min. The measures were read in millimeters of wetting after 5 min. The healthy volunteers were subjected to an ocular surface examination to ensure that pathologies associated with the ocular surface were not present, as well as the absence of allergic or atopic history, as required for donation.

### 4.2. Antibody Microarray Validation and Analysis of Pathological Samples

**Figure 7:**
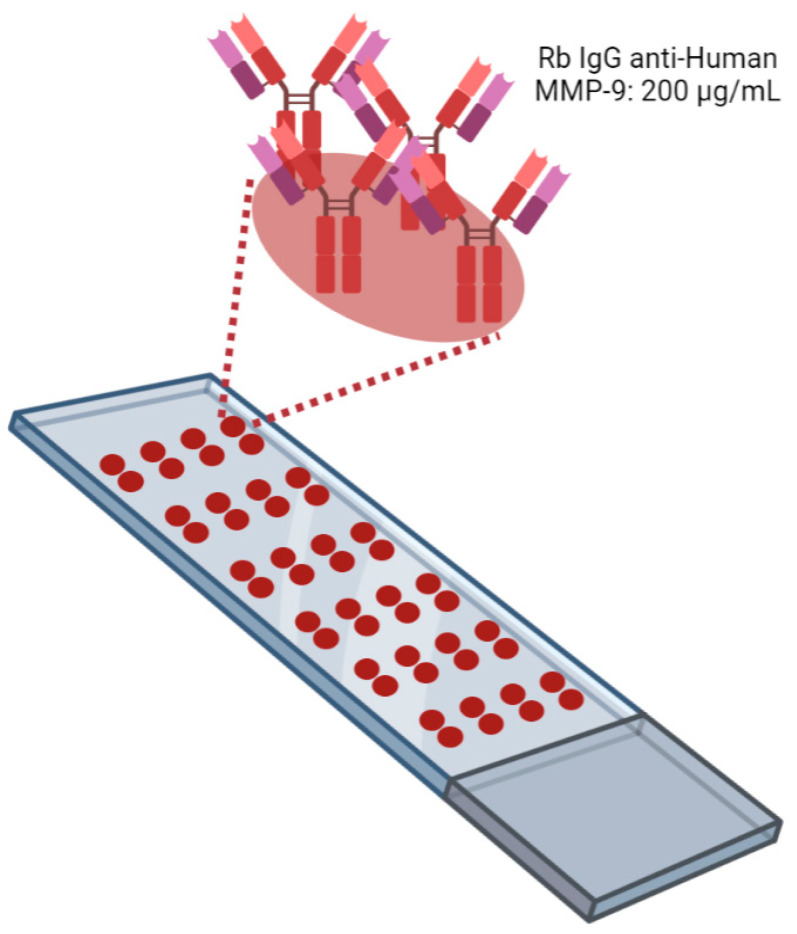
Schematic representation of a microscope glass slide with AbMAs printed. Twenty-four AbMAs with two spots of rabbit IgG anti-human MMP-9 at 200 µg/mL in SIVG 0.05% were immobilized onto treated slides. Image created with BioRender.com.

The AbMAs were fabricated as follows. Microscope glass slides of 76 × 26 mm with 45° frosted ends purchased from LineaLAB (#1053057, Badalona, Spain) were preactivated with an acid treatment carried out following different washing steps to make the surface hydrophobic (EP2048534A4, IMG Pharma Biotech S.L., Derio, Spain). Twenty-four antibody-microarrays (AbMAs) were printed onto each slide using a four-column-six row format (Figure 7). Each AbMA had two replicate spots of rabbit IgG anti-human MMP-9 (#10327-R043, Sino Biological, Beijing, China) immobilized at 200 µg/mL onto SIVG printing solution at 0.05% (IMG Pharma Biotech S.L., Derio, Spain). One drop of 30 nL was printed for each spot using a non-contact microarrayer Nano_plotter (NP 2.1., GeSiM mbH, Radeberg, Germany). The AbMAs were printed on each slide under controlled humidity (60%) at room temperature (RT) and were stored at −20 °C until usage. Four slides of 24 AbMAs were immobilized on each batch printing. Four batch printings were carried out.

These AbMAs were used first for the characterization of the eight tear samples from healthy controls, in which the concentration of MMP-9 was determined before using an ELISA kit versus human MMP-9 from R&D Systems (#DMP900, Minneapolis, MN, USA). Secondly, they were used for quantifying tear MMP-9 in the 20 pathological samples. The immunodetection protocol for tear MMP-9 detection was performed as follows. Slides were firstly thawed and dried for 30 min at RT in a drying chamber. Then, they were washed thrice for 5 min with phosphate buffer saline with tween at 0.01% (0.01% PBS-T) in a slide mailer in agitation, and AbMAs were incubated with blocking solution (milk powder at 2.5% in 0.01% PBS-T) for 10 min at RT. The blocking solution was washed with distilled water, and slides were dried with a fan for 10 min. AbMAs were incubated overnight at 4 °C in a slide humidity chamber with the sample diluted at 1:10 in 0.5% PBS-T with sodium dodecyl sulfate (SDS, #436143, St. Louis, MA, USA) at 0.01%, or MMP-9 (#10327-HNAH, Sino Biological, Beijing, China) at the desired concentration for developing the calibration line/curve. A final volume of 20 µL was used for each AbMA. After incubation, slides were washed twice with 0.5% PBS-T and once with 0.01% PBS-T for 10 min each in a slide mailer in agitation. Then, slides were dried with a fan for 10 min, and AbMAs were incubated for 1 h with primary antibody mouse IgG anti-human MMP-9 (#10327-MM01, Sino Biological, Beijing, China) at 1.25 µg/mL in blocking solution at RT in a slide humidity chamber. After incubation, slides were washed once with 0.5% PBS-T and twice with 0.01% PBS-T for 5 min each in a slide mailer in agitation. Then, slides were dried with a fan for 10 min, and AbMAs were incubated for 1 h with secondary antibody goat IgG anti-mouse IgG conjugated with Alexa Fluor 555 (#ab150118, Cambridge, UK) at 1.25 µg/mL in blocking solution at RT in a slide humidity chamber. Finally, slides were washed once with 0.5% PBS-T, twice with 0.01% PBS-T, once with PBS1X, and once with distilled water for 5 min each in a slide mailer in agitation. Slides were dried with a fan, and the fluorescent signal was revealed using a ChemiDoc Imaging System: Universal Hood 3 (BioRad, Hercules, CA, USA) with Green EPI laser illumination and a 605/650 nm filter.

The signal was quantified using the software ImageScanner (IMG Pharma Biotech S.L., Derio, Spain). Simple bivariate correlation was calculated when comparing ELISA and AbMA quantifications, setting the significance at α = 0.05 using a two-tailed test for validating the reliability of the test. When assessing the differences between pathological groups, firstly, a Shapiro–Wilk test was carried out for testing normality setting and the significance at α = 0.05 using a two-tailed test. The Cliff’s delta value was calculated for quantifying the amount of difference between groups. Data were processed with GraphPad Prism 9.2.0 (GraphPad Software, San Diego, CA, USA). Statistical analyses were carried out with the software SPSS 23.0 (IBM, Armonk, NY, USA).

### 4.3. Evaluation of the Diagnostic Ability of the Test

In order to assess different parameters of the test, two analyses were carried out. An ROC curve analysis was performed, aiming to determine the best detection threshold using our data; additionally, a confusion matrix was set up in order to calculate sensitivity, specificity, and accuracy. Data were processed using Excel 360 spreadsheet software (Microsoft, Redmond, WA, USA).

## 5. Conclusions

A customized AbMA test was fabricated for tear MMP-9 quantification in human samples, managing to detect the biomarker in pathologies that involve inflammation of the ocular surface, such as cataracts, glaucoma, meibomian gland dysfunction, allergy, or dry eye. The test was firstly validated through comparison with the gold standard ELISA and, after its usage for pathological sample characterization, the optimal pathological detection threshold was calculated using an ROC curve, and sensitivity, specificity, and accuracy parameters were estimated through a confusion matrix. With these data, we confirmed the reliability of our AbMA test for the quantification of MMP-9 concentration in human tear samples. The use of biomarker detection technologies as a predictor in the diagnosis of inflammatory ocular pathologies will be useful also in evaluating the prognosis and for monitoring the progression of the pathology and the response to treatments. These will ease the performance of the ophthalmologist, resulting in a greater improvement in patients’ health.

## Figures and Tables

**Table 1:** Baseline characteristics of the patients and healthy control individuals. The healthy controls were collected from volunteers without any ocular pathology diagnosed.

Tear Sample	Group	Age	Gender	Conditions	Schirmer’s Test (mm)
HC 1	Healthy Control	25	Male	n/a	n/a
HC 2	Healthy Control	26	Female	n/a	n/a
HC 3	Healthy Control	30	Female	n/a	n/a
HC 4	Healthy Control	23	Female	n/a	n/a
HC 5	Healthy Control	40	Male	n/a	n/a
HC 6	Healthy Control	23	Female	n/a	n/a
HC 7	Healthy Control	24	Female	n/a	n/a
HC 8	Healthy Control	29	Female	n/a	n/a
P 1	Patient	79	Female	Cataracts	5
P 2	Patient	73	Female	Cataracts	5
P 3	Patient	66	Female	Cataracts	3
P 4	Patient	81	Female	Cataracts	5
P 5	Patient	89	Female	Cataracts	2
P 6	Patient	62	Female	Cataracts	0
P 7	Patient	70	Male	Cataracts	1
P 8	Patient	73	Female	Cataracts	3
P 9	Patient	68	Female	Glaucoma	6
P 10	Patient	60	Male	Glaucoma	5
P 11	Patient	75	Female	Glaucoma	7
P 12	Patient	70	Female	Glaucoma	4
P 13	Patient	82	Male	Glaucoma	5
P 14	Patient	82	Male	Glaucoma	5
P 15	Patient	52	Male	MGD	5
P 16	Patient	49	Female	Allergy	4
P 17	Patient	29	Female	DE	5
P 18	Patient	30	Female	DE	6
P 19	Patient	49	Female	Allergy	5
P 20	Patient	38	Female	MGD + DE	3
